# Potential Early Predictors for Outcomes of Experimental Hemorrhagic Shock Induced by Uncontrolled Internal Bleeding in Rats

**DOI:** 10.1371/journal.pone.0080862

**Published:** 2013-11-26

**Authors:** Zaid A. Abassi, Marina Okun-Gurevich, Niroz Abu Salah, Hoda Awad, Yossi Mandel, Gadi Campino, Ahmad Mahajna, Giora Z. Feuerstein, Mike Fitzpatrick, Aaron Hoffman, Joseph Winaver

**Affiliations:** 1 Department of Physiology and Biophysics, Faculty of Medicine, Technion, Haifa, Israel; 2 Research Unit, Rambam Medical Center, Haifa, Israel; 3 Israeli Medical Corps, Tel Aviv, Israel; 4 The Mina & Everard Goodman Faculty of Life Sciences, Bar Ilan University, Ramat Gan, Israel; 5 Department of General Surgery, Rambam Medical Center, Haifa, Israel; 6 Cellphire Inc. Rockville, Maryland, United States of America; 7 Department of Vascular Surgery, Rambam Medical Center, Haifa, Israel; Emory University, United States of America

## Abstract

Uncontrolled hemorrhage, resulting from traumatic injuries, continues to be the leading cause of death in civilian and military environments. Hemorrhagic deaths usually occur within the first 6 hours of admission to hospital; therefore, early prehospital identification of patients who are at risk for developing shock may improve survival. The aims of the current study were: 1. To establish and characterize a unique model of uncontrolled internal hemorrhage induced by massive renal injury (MRI), of different degrees (20-35% unilateral nephrectomy) in rats, 2. To identify early biomarkers those best predict the outcome of severe internal hemorrhage. For this purpose, male Sprague Dawley rats were anesthetized and cannulas were inserted into the trachea and carotid artery. After abdominal laparotomy, the lower pole of the kidney was excised. During 120 minutes, hematocrit, pO_2_, pCO_2_, base excess, potassium, lactate and glucose were measured from blood samples, and mean arterial pressure (MAP) was measured through arterial tracing. After 120 minutes, blood loss was determined. Statistical prediction models of mortality and amount of blood loss were performed. In this model, the lowest blood loss and mortality rate were observed in the group with 20% nephrectomy. Escalation of the extent of nephrectomy to 25% and 30% significantly increased blood loss and mortality rate. Two phases of hemodynamic and biochemical response to MRI were noticed: the primary phase, occurring during the first 15 minutes after injury, and the secondary phase, beginning 30 minutes after the induction of bleeding. A Significant correlation between early blood loss and mean arterial pressure (MAP) decrements and survival were noted. Our data also indicate that prediction of outcome was attainable in the very early stages of blood loss, over the first 15 minutes after the injury, and that blood loss and MAP were the strongest predictors of mortality.

## Introduction

 Uncontrolled hemorrhage, resulting from traumatic injuries, continues to be the leading cause of death in civilian (accounting for 40% of total deaths) and military (accounting for 50% of total deaths) environments [1-3]. According to US military reports, 15-30% of all deaths are potentially preventable, and of those 80-87% are deaths occurring from hemorrhage [4,5]. 

 Hemorrhagic deaths usually occur very early, within the first 6 hours of admission to hospital [1,6]. Hence, early identification of patients who are at risk for developing shock and death is imperative in order to maximize effective treatments to preserve vital cardiovascular and metabolic function that favorably impact morbidity and mortality. Modern treatment of hemorrhagic shock includes control of bleeding such as bandages, direct pressure, tourniquets or hemostatic dressings and restoration of intravascular volume using crystalloids, colloids or blood products. Several parameters were proposed as indicators of the patient's condition after traumatic hemorrhage and currently used as end-points of resuscitation: mean arterial pressure (MAP), heart rate, urine output, cardiac index, oxygen consumption, oxygen delivery, base excess (BE) or base deficit, lactate, and mucosal gastric pH [7]. In contrast, uncontrolled non-compressible severe hemorrhage from internal organs, usually occurs following injuries to the thoraco-abdominal cavity. The liver and the spleen are the most commonly injured abdominal organs [8], therefore, during the last century, models of splenic and hepatic injuries in large and small laboratory animals were developed for studying of various aspects of uncontrolled hemorrhage. However, in preliminary experiments conducted in our laboratory using models of splenic injuries, we found that maximal hemorrhage volume in these models did not exceed 25% of calculated total blood volume. According to Coburn, injury to the kidneys occurs in up to 10% of all abdominal injuries [9], thus in search for models of uncontrolled hemorrhage reaching high bleeding volumes we embarked on developing the model of partial ablation of the kidney. This model was used previously in studies aimed at developing hemostatic agents, and was applied mostly in heparinized rats [10-12]. In this regards, we attempted to characterize this model over a non-heparinized background. The unheparinized bleeding model has few advantages over the heparinized one. While the former is characterized by spontaneous gradual bleeding resembling the natural course of hemorrhagic shock of causalities in the battle field, the heparinized model is associated with intensive and often fatal bleeding. Moreover, the unheparinized model is suitable for examining potential anticoagulant therapies, whereas the heparinized one may interfere with efficacy of these agents, thus masking their real hemostatic properties.

Our first objective was to characterize graded bleeding paradigms (generated by differential loss of kidney mass). The second objective aimed at assessment of various hemodynamic and biochemical biomarkers as predictors for mortality outcome. This topic is of special interest since the traditionally used parameters, such as blood pressure and heart rate, may mask activation of compensatory mechanisms and do not necessary reflect the severity of the traumatic injury. Moreover, late identification of massive internal bleeding may lead to transportation of these patients to inadequately equipped medical center and consequently delayed treatment. Therefore, we compared the usefulness of selected hemodynamic and biochemical parameters in the early prediction of the outcome after MRI model. 

## Materials and Methods

### Animals

Studies were conducted on male Sprague Dawley rats, weighing 320- 350 g, purchased from Harlan Laboratories, Jerusalem, IL. Rats were maintained on standard rodent chow and water ad libitum and housed in regular cages (3 animals per cage) in the Experimental Animals Facility of the Faculty of Medicine, Technion, IIT Haifa, Israel, on 12:12 light-dark cycle. The animals were fasted for 4 hours prior to the experiment. The current study was approved and conducted according to the guidelines of the Institutional Animal Care and Use Committee of the Technion, Faculty of Medicine (1997). 

### Experimental groups

The rats were divided into 4 groups and randomized to one of the 4 nephrectomy procedures: 20% nephrectomy (n=19), 25% nephrectomy (n=16), 30% nephrectomy (n=12), and 35% nephrectomy (n=12). The percentage was calculated by weighing the excised pole and the remaining in situ part of the kidney at the end of the experiment.

### Surgical preparation

On the day of the experiment, rats were anesthetized by Inactin (thiobutabarbital sodium; Sigma Chemicals, 100 mg/kg, i.p.) and placed on a thermo-regulated surgical table that maintains body temperature at 37°C. Following tracheotomy, polyethylene catheter (PE-50) was inserted into the left carotid artery for blood pressure and heart rate monitoring. A mid-abdominal laparotomy section was opened to allow access to the organ to be injured. 

### Renal injury model

Renal ablation was performed in principle by surgical removal of the lower pole of the left kidney as previously described [11,12], and was adopted in our study with the exception of heparinization. The exposed lower pole of the left kidney was transected inferior to the renal pelvis and hilum (20%-35%) of the kidney mass as needed, and the kidney allowed free bleeding into the peritoneal cavity. 

### Monitoring of hemodynamic and biochemical variables

After completing the partial nephrectomy, the abdominal cavity was closed and the animals were continuously monitored for a maximum of 120 min or death as assessed by lack of respiratory movement. The animals remained fully anesthetized for the entire duration of the study, up to and including death, as a result of either hemorrhage or euthanasia. The laparotomy incision was then reopened and free intraperitoneal cavity clotted and unclotted blood was collected on pre-weighed sterile pieces of cotton. The amount of absolute blood loss was determined by difference in wet and dry weights. The percentage of blood loss was determined by calculation of blood loss from estimated total blood volume (TBV) of the rat, according to the formula: TBV≅ Rat Weight x 0.06 [13,14]. MAP was measured continuously by pressure transducer (model 1050.1, UFI, Morro Bay, CA, USA) connected to the carotid arterial line, at times -10 (before nephrectomy), 0 (the first minute after partial nephrectomy), 5, 10, 15, 30, 60 and 120 min after the injury. Hematocrit, pO_2_, pCO_2_, BE, potassium, lactate and glucose were determined in blood samples that were withdrawn into glass capillary tubes at -10, 5, 10, 15, 30, 60, 120 min after injury by GEM Premier 3000 ( Instrumentation Laboratory, Lexington, USA). To avoid major changes in the status of intravascular fluid, equal volume of saline was given intra arterially to replace the withdrawn blood samples. At the end of the experiment, the animals were sacrificed by intravenous injection of KCl. 

### Statistical analysis

The results are presented as mean±SEM. Each variable was considered as possible predictors of the changes along time expressed as first and also second order differences between measurements at consecutive time point. In order to find the best predictors we applied the CART (Classification and Regression Tree) method [15]. CART is a flexible non-parametric modern statistical technique that has been adopted for data mining. It is suited for both exploring and modeling the relationship between a response variable and a set of explanatory variables. Moreover, CART enables to detect high order interactions [15]. RPART procedure was used for this analysis (with the software R package version 3.1-50). ANOVA and t-tests were used to assess the significance of the predictors detected by CART. Effect size was computed using Cohen's d statistic.

## Results

Table **1** summarizes the number of rats and extent of injury in each group. 

**Table 1:** Summary of rat groups.

	**Expected Nephrectomy**	**Actual Average Nephrectomy**
**Group 1 (n=19)**	20%	19.1±0.5%
**Group 2 (n=16)**	25%	24.8±0.3%
**Group 3 (n=12)**	30%	29.0±1.0%
**Group 4 (n=12)**	35%	37.0±0.9%

**Figure 1:**
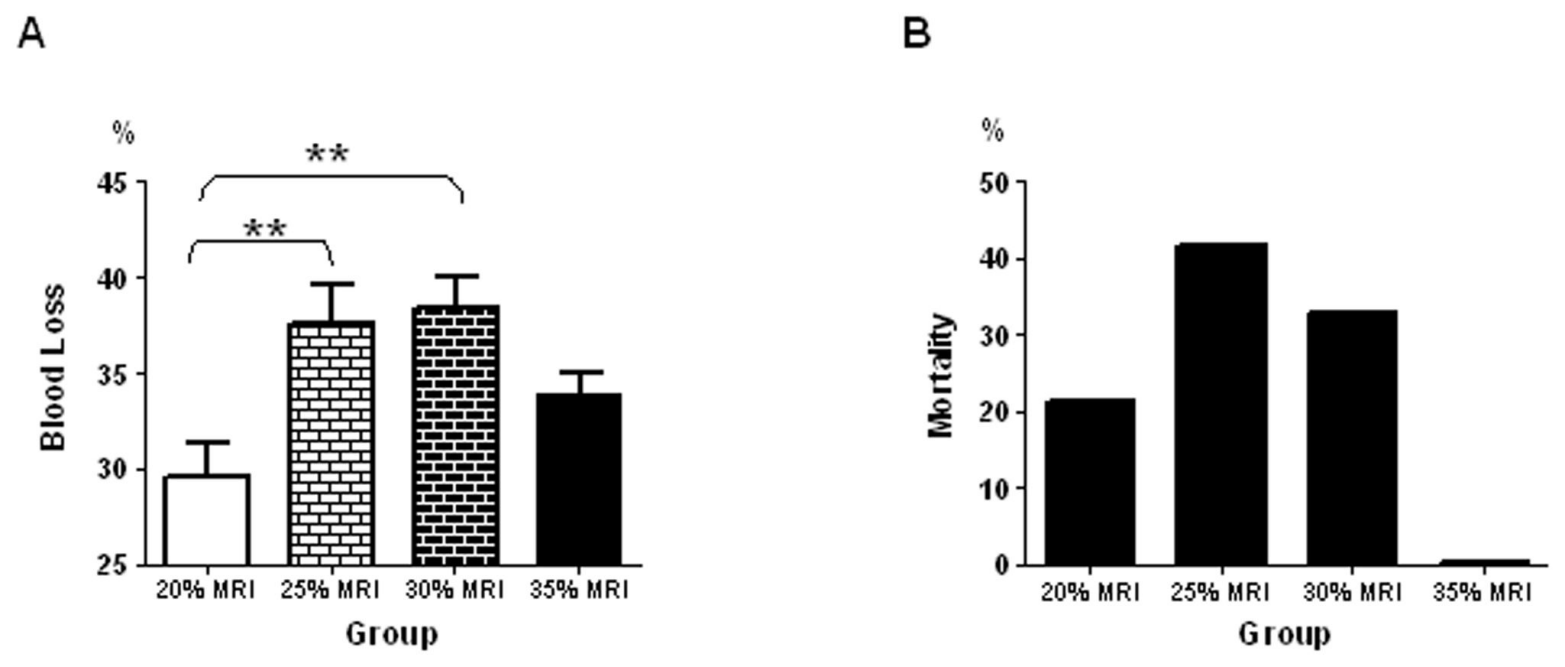
Group Characterization. A) Blood loss in each group, presented as percentage of total blood volume. B) Mortality rate in each experimental group.

According to our results, the highest blood losses were observed in the groups with 25% and 30% nephrectomy and were 37.6±2.1% and 38.4±1.6% of TBV, respectively. Higher degree of nephrectomy (~35% nephrectomy) did not exacerbate blood loss (Figure **1A**). The body weight was decreased proportionally to the blood loss (data not shown). 

During the experiment, 12 out of 59 rats did not survive the entire observation period. The highest mortality rates were observed in 25% and 30% nephrectomy groups, and were 42% and 33% respectively (Figure **1B**). In a pilot study, we found no differences in heart or kidney weight between animals that underwent various degree of nephrectomy and their normal controls. 

### Hemodynamic measurements

**Figure 2:**
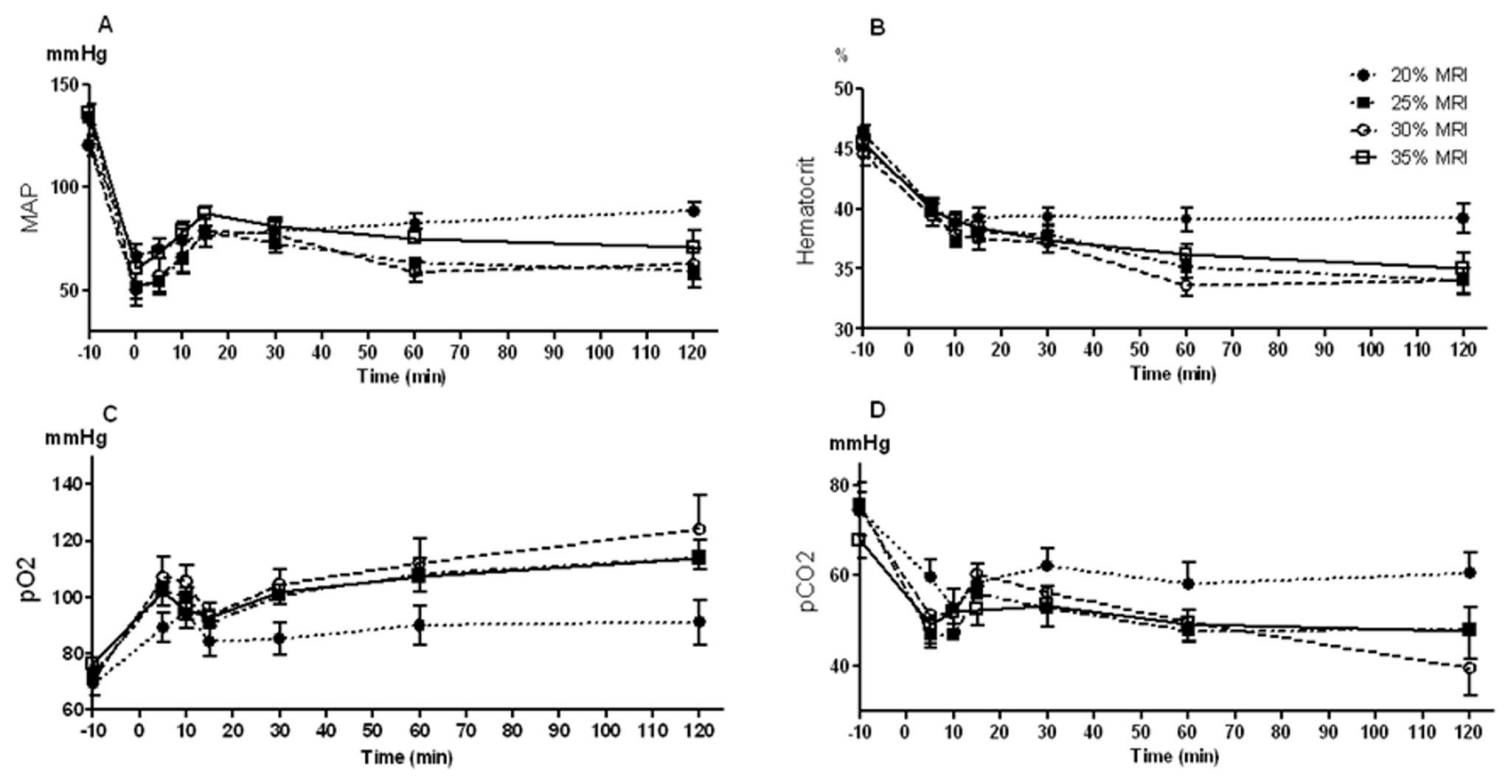
Hemodynamic, hematologic and blood gases measurements during the experiment. A) Mean arterial pressure measured via arterial line. Parameters measured from blood samples: B) Hematocrit, C) Partial O2 pressure, D) Partial CO2 pressure.

The baseline MAP of rats, measured at time point -10 (T_-10_), was comparable in all groups (122-138 mmHg). Immediately after the removal of the lower pole of the kidney, bleeding was initiated and MAP decreased. The lowest MAP measured immediately after the nephrectomy (during the first minute of bleeding) was marked as time point 0 (T_0_). The most prominent decrease was observed in the 25% MRI group, in which the MAP decreased to 43.88±7.45 mmHg. Approximately 5 minutes after the nephrectomy, MAP began to rise, however in none of the experimental groups it reached baseline MAP values. At T_15_ the MAP of all groups was elevated to approximately 80 mmHg. In the 20% MRI group, blood pressure continued to rise slowly, reaching 88.88±7.4 mmHg at the end of the experiment. However, in all other groups, MAP values gradually decreased till the end of observation period reaching values of 56-70 mmHg (Figure **2A**).

### Blood biomarkers

Rats' baseline hematocrit values were 44.9%-45.6%. At the beginning of the bleeding, rapid decreases in hematocrit level were observed until T_10_. At this time point, in 20% MRI group hematocrit values were stabilized at around 39%, and did not change till the end of follow up period. In all other groups, hematocrit values continued to decrease gradually till the end of observation period reaching values of 33.1-34% (Figure **2B**).

Baseline partial O_2_ pressure (pO_2_) in rats was 69.3-80.9 mmHg, and partial CO_2_ pressure (pCO_2_) was 69.1-76.3 mmHg. During the first 5 min of bleeding, the pO_2_ increased, and pCO_2_ decreased. At T_10_ and T_15_, additional decline in pO_2_ was observed, followed by subsequent increase in pO_2_ till the end of observation period. Except for the 20% MRI group, in which oscillations in pCO_2_ were observed during the entire experiment, the pattern of pCO_2_ was the opposite of pO_2_ – after the decrease observed during 5 minutes, there was an increase in pCO_2_ followed by a subsequent decrease (Figures **2C and 2D**).

**Figure 3:**
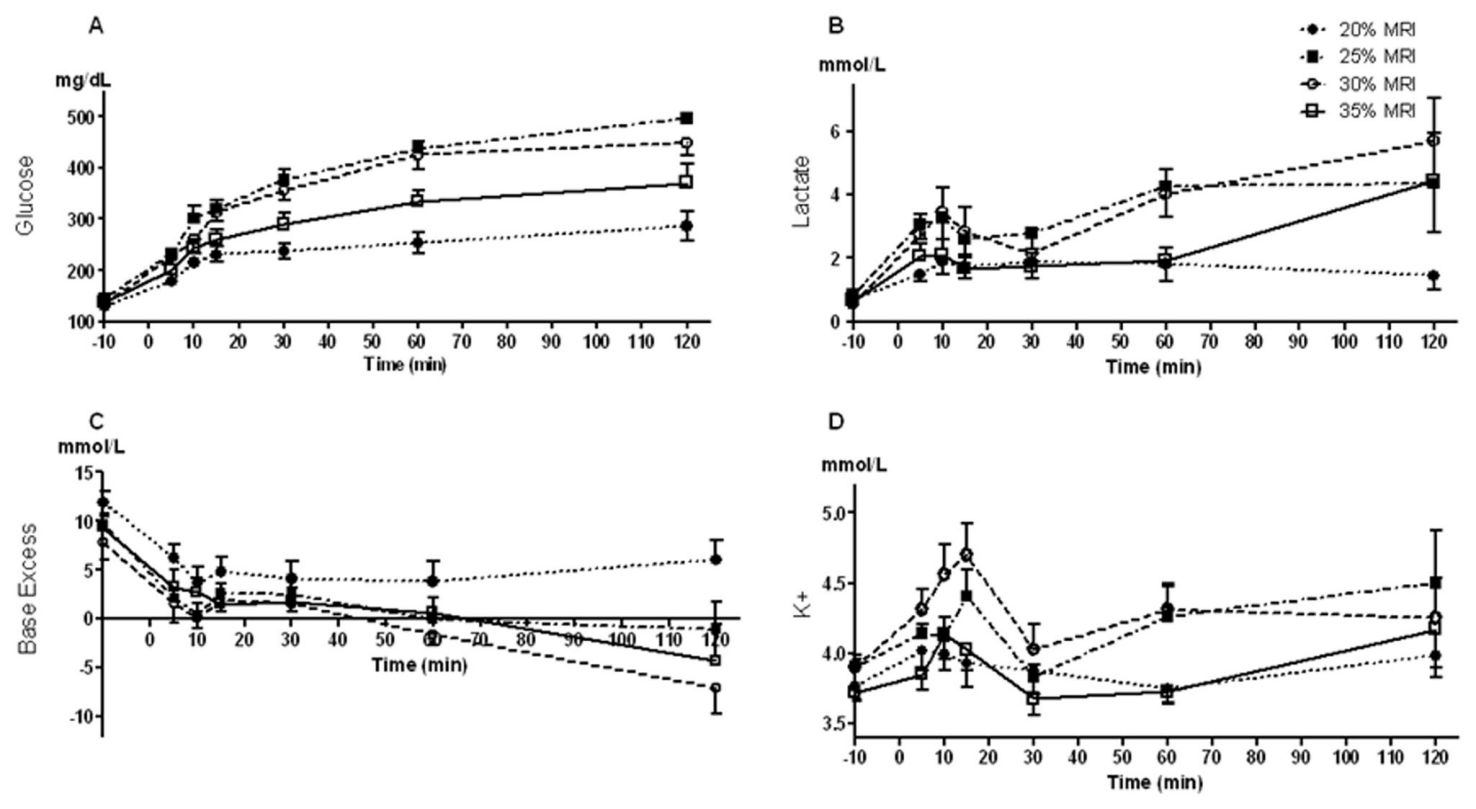
Metabolites, potassium and acidity measurements taken from blood samples. A) Glucose, B) Lactase, C) Base excess, D) Blood potassium.

Baseline blood glucose level in rats' was 134-138.3 mg/dL. After the nephrectomy, the glucose values in all rats increased, and continued to escalate until the end of observation period, where maximal glucose level of ~500 mg/dL was observed in rats with MRI due to 25% nephrectomy (Figure **3A**). 

Baseline lactate level in rats was 0.5-0.7 mg/dL. In 25%, 30%, and 35% MRI groups, blood lactate level increased during the first 10 minutes of bleeding, after which a decline in lactate level was observed, followed by subsequent increase to 4.3-5.7 mg/dL at the end of the experiment. In 20% MRI group, an elevation of lactate values was observed during the first 10 minutes of bleeding, and was followed by subsequent oscillations. The final lactate level in this group was 1.81±0.8 mg/dL (Figure **3B**).

Baseline base excess (BE) values were 7.8-12.5 mml/L. In 20% MRI group, although a reduction in BE was observed during the bleeding, BE level did not reach negative values. In all other groups, a reduction in BE was observed during the first 10-15 minutes of the bleeding. This reduction was followed by further aggravation in BE, resulting severe negative values until the end of observation period (Figures **3C**).

Baseline blood potassium values were 3.68-3.91 mmol/L. During the first 10-15 minutes of bleeding, an elevation of blood potassium level was observed, followed by a decrease, and subsequent increase in potassium values in all groups (Figure **3D**). 

### Correlations of fixed parameters

Statistical examination revealed weak correlations between the extent of nephrectomy and blood loss (r=0.26) or MAP decrease at T_0_ (r=0.23). However, significant correlation between blood loss and MAP decrease at T_0_ was found (P<0.005).

In addition, correlations between rats' survival at the end of the experiment and blood loss (32.7±1% blood loss in survived group versus 41.2±1.9% blood loss in non-surviving group, P<0.005) or MAP decrease at T_0_ (49.6±2.7% drop in surviving group versus 70.9±4.6% drop in non-survived group, P<0.001) were found. All correlations are summarized in Table **2**.

**Table 2:** Summary of all correlations.

	**P value**	**r**
**% Blood loss vs. % Nephrectomy**	0.05	0.26
**% MAP decrease vs. % Nephrectomy**	0.08	0.23
**% MAP decrease vs. % Blood loss**	<0.005	0.4
**% MAP decrease vs. Mortality**	<0.001	
**% Blood loss vs. Mortality**	<0.005	

### Prediction of outcome using mortality

Initially, we chose rats' survival at the end of the experiment to define the severity of rats' condition after MRI, and used it as the dependent variable. By using the MAP and different parameters measured in blood as independent variables, we built models for predicting rats' survival. 

When MAP or pO_2_ were used as the independent variables, T_10_ was determined as the earliest possible prediction of survival. At this point, if MAP was 51.5 mmHg or higher, or if pO_2_ was lower than 105.5 mmHg, rats would survive. 

When hematocrit, lactate or potassium levels were used as the independent variables, the earliest possible prediction of survival was determined at T_15_. At this point, if hematocrit was 35 or higher, or if lactate was lower than 4.4 mmol/L, or if potassium was lower than 4.85 mmol/L, rats would survive. 

 BE prediction was based on two variables: T_15_ and the difference between T_10_ and T_5_. At T_15_, rats that have BE of -2.95 mmol/L or higher would survive. Otherwise, if the difference in BE values between T_10_ and T_5_ is -1.45 mmol/L or higher, the prediction determines that these rats will also survive. 

The specificity and sensitivity for models based on MAP, lactate, potassium and pO_2_ pressure were higher than 50%, while the highest odds ratio was observed in the model predicting survival by MAP (3.78, 95%CI 2.89-4.68, Table **3**). 

**Table 3:** Summary of all parameters predicting mortality following uncontrolled hemorrhagic shock.

**Parameter**	**Time-point**	**Condition**	**OR**	**95% CI**	**Specifity [%]**	**Sensitivity [%]**	**PPV [%]**	**NPV [%]**
**MAP**	10	<51.5 mmHg	3.78	2.89-4.68	93.6	75%	75	93.6
**Lactate**	15	>=4.4 mmol/L	3.43	2.5-4.36	95.7	58.3	77.8	89.8
**PO_2_**	10	>=105.5 mmHg	2.8	2.02-3.57	89.1	66.7	61.5	91.1
**BE**	15	<-2.95 mmol/L	2.48	1.56-3.41	92.3	50	57.1	90
	10, 5	Δ(T_10_-T_5_)<-1.95 mmol/L						
**K+**	15	>=4.85 mmol/L	2.44	1.69-3.19	89.1	58.3	58.3	89.1
**Hct**	15	<34.5%	2.3	1.47-3.14	93.3	41.7	62.5	85.7

Prediction was based on parameters at three time points: 5, 10, and 15 minutes after the injury. At each time point the values of the parameter were arranged into a condition. Fulfillment of this condition, predicts mortality. The parameters in the table are arranged from the highest to the lowest OR. OR – Odds ratio (OR>1, predicts mortality), CI-Confidence Interval, PPV-Positive predictive value [# identified as dead while dead/(# identified as dead while dead + #identified as dead while survived)] , NPV – Negative predictive value [# identified as survived while survived/(# identified as survived while survived + #identified as survived while dead)]. Specifity – [#identified as survived while survived/(identified as survived while survived + #identified as dead while survived)]. Sensitivity – [#identified as dead while dead/(identified as dead while dead + #identified as survived while dead)]

### Prediction of outcome using amount of blood loss

At the second stage, we chose blood loss to define the severity of rats' condition after MRI, and used it as the dependent variable. By using the MAP and various parameters measured in blood as independent variables, we built models predicting blood loss. 

When MAP was used as the independent variable, T_5_ was determined as the earliest possible determination of blood loss amount. At this point, if MAP was 32.5 mmHg or higher, rats would lose 33.45±7.33% of TBV, otherwise rats would lose 41.54±5.88% of TBV.

When lactate was used as the independent variable, the earliest possible estimation of blood loss amount was determined at T_10_. At this point, if lactate was lower than 4.15 mmol/L, rats would lose 33.16±7.45% of TBV, otherwise they would lose 39.12±6.03% of TBV. 

The earliest possible predictions by glucose or potassium were able at T_15_. At this point if glucose was lower than 249.5 mg/dL, rats would lose 29.56±7.5% of TBV, otherwise they would lose 36.72±6.55% of TBV. If potassium was lower than 4.85 mmo/L, rats would lose 33.15±7.28% of TBV, otherwise they would lose 40.16±5.89% of TBV. 

The highest effect size of all models was found in the model predicting blood loss by MAP (1.124, Table **4**), making MAP the best parameter for blood loss prediction. 

**Table 4:** Summary of all parameters predicting blood loss following uncontrolled hemorrhagic shock.

**Parameter**	**Time-point**	**Condition**	**Group 1**	**Group 2**	**Effect Size**
**MAP**	5	>=32.5 mmHg	33.45±7.33	41.54±5.88	1.124
**Glucose**	15	<249.5 mg/dL	29.56±7.5	36.72±6.55	1.043
**K+**	15	<4.85 mmo/L	33.15±7.28	40.16±5.89	0.996
**Lactate**	10	<4.15 mmol/L	33.16±7.45	39.12±6.03	0.834

Prediction was based on parameters at three time points: 5, 10, and 15 minutes after the injury. For each predictor the data was divided into 2 groups: group 1, predicting lower blood loss and group 2, predicting higher blood loss. Fulfillment of the condition for each parameter predicts blood loss of group 1, otherwise the prediction is blood loss of group 2. Effect size >0.8 – large effect between the groups.

## Discussion

One of the goals of the current study was to establish and characterize a model of MRI of different degrees in a rat. In this model, the lowest blood loss and mortality rate were observed in the group with 20% nephrectomy. Elevation in the extent of partial nephrectomy to 25% and 30% significantly increased blood loss and mortality rate in these groups. However, further increase in partial nephrectomy did not aggravate blood loss and mortality. In our study, changes in MAP were observed immediately after kidney ablation. A positive correlation was found between the blood loss and the initial decline in MAP, i.e., animals that displayed severe hypotension in the first 5 minutes, exhibited the highest degree of blood loss.

After partial nephrectomy, changes in different biomarkers were observed. As noticed, these changes can be divided into two phases: the primary phase, occurring during the first 15 minutes after the induction of bleeding, and the secondary phase, beginning 30 minutes after the induction of bleeding and lasting throughout the observation period. These two phases were also observed in the MAP measurements during the experiment. Since most of the recent studies of uncontrolled hemorrhage in a rat models focused on developing anti-bleeding therapies, they did not detect the two phases described by us during the 120 minutes after the induction of bleeding. The main reason is the design of these studies: most of them did not record MAP or other biomarkers during the first 15 minutes after the bleeding, or administrated resuscitation fluids immediately after the beginning of bleeding, thus affecting the physiological response [14,16,17]. However, in studies using a swine model of uncontrolled hemorrhage, the same pattern of MAP response was observed in untreated animals during 120 minutes [18,19]. In our study, the most substantial changes in MAP and other parameters were observed during the primary phase. Hence, the importance of detecting these two stages of uncontrolled bleeding is that we were able to predict the outcome based on the measurements collected parameters during the initial 15 minutes.

When comparing the hemodynamic and metabolic parameters measured in our study, to those measured in studies using the massive splenic injury model [13,14], we noticed that hemodynamic and metabolic deterioration after spleen injury were less severe, compatible with the results of preliminary study in our lab on splenic injury model (data not shown). In 2009, Warner et al., compared three models of uncontrolled bleeding: liver, spleen and kidney injury. He found that the highest blood loss occurred in the model of kidney injury, due to its large wound surface [20]. This finding supports the use of kidney injury model for studies of extensive uncontrolled bleeding.

In our study, 12 out of 59 rats did not survive 120 minutes after the injury (average survival time was 107 minutes). Conversely, in studies using heparinized MRI model, mortality rate was higher and the survival time of untreated animals was much shorter than 30 minutes or less [10,11]. Therefore, although the use of heparinized model may be applied for studies of hemorrhagic shock agents, it does not represent the clinical situation of the majority of the trauma victims. When cardiac output (CO) was measured in preliminary experiments in rats with 30% nephrectomy, we detected a gradual decline throughout the experiment, reaching undetectable values prior to death in those who did not survive the hemorrhagic shock (data not shown). These findings suggest that the main reason for mortality in this model of MRI is cardiac arrest. However, shock may lead to hypoxemia, which is known to induce cardiac arrest. Due to the short term nature of the current protocols, we did not detect any changes in heart or lung weight normalized to body weight, suggesting that cardiac rather than pulmonary congestion is the main suspect for death in this model. Additional studies are required in order to examine whether MRI model is associated with lung morphological changes, which do not necessary lead to alterations in lung weight.

Surprisingly, we did not detect deterioration in hemodynamic and biochemical parameters, nor in the mortality and blood loss rate, when expanding the injury to 35% nephrectomy. We assume that this might be due to: 1) The proximity of the site of incision to the renal artery, where the intensity of vasoconstriction response is stronger than in smaller vessels; 2) The blood clot formed in wounded large vessels is more stable, perhaps because it contains more coagulation factors in comparison to smaller vessels; 3) The smaller surface area of the vasculature in nephrectomy of 35%. An alternative approach to reach 35% nephrectomy is resection of the kidney at both poles (anterior and posterior). Another way to achieve 35% percent nephrectomy might be by performing a longitudinal cut of the kidney. 

Trying to determine the best markers to identify animals with the worst condition, we defined the severity of rats' condition by two representatives: one using rats' mortality, and the second using the extent of blood loss. These representations allowed us to build statistical models in order to predict the outcome after MRI. Accordingly, during the first 15 minutes after the beginning of bleeding, MAP was found to be the best predictor of outcome by both mortality and blood loss. This is in contradiction to previous reports, concluding that hemodynamic markers are unreliable indicators of patient's condition, especially in compensated shock [7,21,22]. The main difference between our study and the majority of previous studies is that the latter were performed on trauma patients (prospectively or retrospectively reviewed), that had MAP measured at hospital admission. During the time passing between the injury and the admission, the MAP could have been elevated due to compensatory mechanisms activated during shock or due to administration of resuscitation fluids. However, a recent study by Arbabi et al., determined that prediction of mortality was possible by both prehospital and emergency department systolic blood pressure [23].

The second best predictor of mortality in our study was lactate. Rats that displayed high lactate levels had remarkable blood loss and higher mortality rate than those that exhibited mild increase in this biomarker. In line with this finding, Guyette et al reported that prehospital measurement of serum lactate in trauma patients predicted mortality, emergent operations and multiple organ dysfunction [24]. Furthermore, previous studies demonstrated that lactate level measured at admission to the hospital is a good predictor of mortality [25-27]. In this context, the duration of hyperlactemia in these patients was associated with mortality and development of multiple organ failure [26,28]. Lactate level also predicted blood loss of nearly 40%. According to ATLS classification of hemorrhage, hemorrhage of 40% and more is considered as preterminal event requiring immediate therapy. Lactate is known as a marker of the decrease in organ perfusion; therefore, high lactate level may serve as a good indicator of tissue hypoxia, even when clinical signs of shock, such as MAP, have been corrected. 

Previous studies showed that admission glucose is a good predictor of mortality [27-29]. In our study, glucose values did not predict mortality, however, glucose values higher than 250 mg/dL predicted more than 35% blood loss. There are several explanations for why glucose level did not predict mortality in our study: first, we specifically measured the prediction of mortality within the first 15 minutes after the injury, whereas other studies referred to admission glucose values. Second, our study included only 59 rats, whereas the previously mentioned studies included more than 450 study subjects, hence it is possible that larger number of rats would have revealed glucose as a predictor of mortality in the current study. 

Several studies, comparing the ability of admission BE (or base deficit) and lactate to predict the outcome after traumatic injuries, have found BE to be superior to lactate in predicting mortality [30,31]. In our study, BE was also found to be a predictor of mortality, yet it was not a better predictor than lactate. Possible explanation is that we followed the deterioration of biochemical markers in untreated animals; conversely, the results of those studies may have been affected by treatment with resuscitation fluids, especially those containing crystalloids, known in their ability to develop hyperchloremic acidosis [32].

Hematocrit in our study was also determined as a predictor for mortality. However, its application in trauma patients is in great doubt, as these patients are usually treated by resuscitation fluids at an early stage, resulting in blood dilution. 

Interestingly, in our study potassium was found to be a predictor of both mortality and blood loss of more than 40%. To our knowledge, there are no previous studies referring to potassium as a predictor of outcome after traumatic hemorrhage. However, a recent study by Rocha Filho, showed that, in swine uncontrolled hemorrhage model, the rate of elevation in serum potassium after bleeding was higher in non-survivors compared to survivors [33]. In addition, they showed that potassium continued to rise in non-survivors even during resuscitation, as opposed to survivors, in which potassium decreased during treatment to normal values. 

According to our data, mortality was also predicted by elevation in pO_2_. This elevation occurred due to hyperventilation, trying to compensate for the decrease in oxygen delivery to the tissues. Two previous studies conducted by Kerger et al., on hamsters [34,35], showed that animals, which did not survive after hemorrhagic shock, had significantly higher arterial pO_2_ levels in comparison to survivors. They also showed that despite the increase in arterial pO_2_ during shock, there was reduction in microvascular pO_2_, due to vasoconstriction and reduction in blood flow in these vessels. 

The main aim of our experimental study was to find early, on-field, predictors of the severity of traumatic injury. It is important that military and civilian paramedic teams would be guided to provide pre-hospital treatment based on reliable markers of injury severity. Another important issue to consider is the availability of the equipment required for measuring those markers. The three best indicators of injury severity presented in our study were MAP, lactate and glucose. MAP measurements can easily be used on the field, since hemodynamic monitoring is routinely carried out by paramedics. The glucose and lactate measurements are possible by using a glucometer, and a handheld, point of care lactate meter [24].

The advantage of our study as opposed to clinical studies on human subjects is that we were able to test and characterize the response of the body to isolated internal bleeding, without the interference of additional factors, such as brain injuries, delay of admission to the hospital, administration of fluids, resuscitation, etc. 

Our study has few potential limitations, such as: 1- the relatively small number of rats; 2- urine was not monitored; 3- We focused on few selected biomarkers, based on previous literature and our own experience. Further research into profiling wide spectrum of biomarkers could potentially identify the severity of hemorrhagic shock. 

## Conclusions

By using an experimental model of internal bleeding in rats, we disclosed two phases of body response to hemorrhage: an early phase during the first 15 minutes after the injury, and the second phase starting 30 minutes after the injury. We showed that prediction of the outcome of uncontrolled internal hemorrhage is possible during the first 15 minutes after injury. The best predictor of blood loss and mortality in this study was MAP. We also demonstrated that lactate and glucose level in blood are powerful predictors of outcome. Moreover, we believe that the applied model of renal injury can serve in future studies as a platform for examining the efficacy of novel or classic anti bleeding agents. Future studies should evaluate how the best obtained early biomarker of the bleeding severity, responds to various therapeutic approaches, such as fluid infusion, plasma and blood transfusion, etc. Further studies are needed to explore the effect of various treatments on the correlation between mortality rate and potential biomarkers/predictors.
